# Telomerase/myocardin expressing mesenchymal cells induce survival and cardiovascular markers in cardiac stromal cells undergoing ischaemia/reperfusion

**DOI:** 10.1111/jcmm.16549

**Published:** 2021-05-05

**Authors:** Rosalinda Madonna, Simone Guarnieri, Csenger Kovácsházi, Aniko Görbe, Zoltán Giricz, Yong‐Jian Geng, Maria Addolorata Mariggiò, Péter Ferdinandy, Raffaele De Caterina

**Affiliations:** ^1^ Department of Internal Medicine McGovern School of Medicine The University of Texas Health Science Center at Houston Houston TX USA; ^2^ Department of Pathology, Cardiology Division University of Pisa Pisa Italy; ^3^ Center for Advanced Studies and Technology –CAST Chieti‐Pescara Chieti Italy; ^4^ Department of Neuroscience, Imaging and Clinical Sciences Chieti‐Pescara and StemTeCh Group “G. d’Annunzio” University Chieti Italy; ^5^ Department of Pharmacology and Pharmacotherapy Semmelweis University Budapest Hungary; ^6^ Pharmahungary Group Szeged Hungary

**Keywords:** adipose tissue‐derived mesenchymal stromal cells, cardiac stromal cells, extracellular vesicles, myocardin, simulated ischaemia‐reperfusion, telomerase

## Abstract

Cardiac stromal cells (CSCs) contain a pool of cells with supportive and paracrine functions. Various types of mesenchymal stromal cells (MSCs) can influence CSCs in the cardiac niche through their paracrine activity. Ischaemia/reperfusion (I/R) leads to cell death and reduction of the paracrine activity of CSCs. The forced co‐expression of telomerase reverse transcriptase (TERT) and myocardin (MYOCD), known to potentiate anti‐apoptotic, pro‐survival and pro‐angiogenic activities of MSCs isolated from the adipose tissue (AT‐MSCs), may increase CSC survival, favouring their paracrine activities. We aimed at investigating the hypothesis that CSCs feature improved resistance to simulated I/R (SI/R) and increased commitment towards the cardiovascular lineage when preconditioned with conditioned media (CM) or extracellular vesicles (EV) released from AT‐MSCs overexpressing TERT and MYOCD (T/M AT‐MSCs). Murine CSCs were isolated with the cardiosphere (CSps) isolation technique. T/M AT‐MSCs and their secretome improved spontaneous intracellular calcium changes and ryanodine receptor expression in aged CSps. The cytoprotective effect of AT‐MSCs was tested in CSCs subjected to SI/R. SI/R induced cell death as compared to normoxia (28 ± 4 vs 10 ± 3%, *P* = .02). Pre‐treatment with CM (15 ± 2, *P* = .02) or with the EV‐enriched fraction (10 ± 1%, *P* = .02) obtained from mock‐transduced AT‐MSCs in normoxia reduced cell death after SI/R. The effect was more pronounced with CM (7 ± 1%, *P* = .01) or the EV‐enriched fraction (2 ± 1%, *P* = .01) obtained from T/M AT‐MSCs subjected to SI/R. In parallel, we observed lower expression of the apoptosis marker cleaved caspase‐3 and higher expression of cardiac and vascular markers eNOS, sarcomeric α‐actinin and cardiac actin. The T/M AT‐MSCs secretome exerts a cytoprotective effect and promotes development of CSCs undergoing SI/R towards a cardiovascular phenotype.

## 1 INTRODUCTION

Heart failure (HF), with or without myocardial ischaemia, is a leading cause of death worldwide.^1^ HF can be attributed, at least in part, to the limited ability of the heart to repair or regenerate the damaged myocardium.^2^ As a result of myocardial injury, dead or malfunctional cells are not replaced with new healthy cells, but instead by acellular, non‐contractile fibrotic tissue. Such replacement initiates a series of subsequent adverse events, such as negative ventricular remodelling, reduced global function and, eventually, clinical manifestations of HF.^3^

Cardiospheres (CSps) are a heterogeneous population of cells with the potential for clinical cell therapy.^4^, ^5^, ^6^ CSps contain a heterogeneous population of residing non‐cardiomyocyte CD45− CD34− CD31− CD105+ stromal cells (CSCs), phenotypically characterized as fibroblasts, pericytes, endothelial cells, smooth muscle cells and mesenchymal stromal cells, with supportive and paracrine functions for cardiomyocytes.^7^ The crosstalk between cardiomyocytes and CSCs plays a fundamental role for repair processes after cardiac damage through the release of growth factors, pro‐angiogenic factors and the regulation of cardiac metabolism.^8^, ^9^, ^10^ Exposure to ischaemia and reperfusion (I/R) accelerates apoptosis and necrosis of cardiomyocytes and CSCs, hampering their crosstalk and repairing functions, and ultimately favours the development of HF.^11^, ^12^, ^13^ Cardiac microenvironments housing CSCs, known as cardiac niches, provide CSCs with regulatory signals, including oxygen tension, essential for their maintenance, proliferation and differentiation.^14^

A number of studies have recently shown that mesenchymal stromal cells (MSCs) of different origins can influence cardiac niche microenvironment through their paracrine activity.^15^, ^16^ The combination of CSCs and MSCs (the so‐called combo‐approach) is an example of a multipronged cell therapy attempt to cardiac repair.^2^, ^3^, ^17^, ^18^ By virtue of their intense paracrine activities, MSCs can influence the phenotype and paracrine activity of CSCs. A pool of such MSCs with paracrine activity has been described in the adipose tissue (AT).^19^, ^20^ AT‐MSCs thus represent an interesting source of cells for cardiac repair, being able to improve heart function in the infarcted area mainly through paracrine action and soluble factors.^3^ As a result, the two cell populations—AT‐MSCs and CSCs—might cooperate if combined,^21^ establishing an interaction mediated by their respective microenvironments, thus providing relevant biological advantages towards cardiac repair. Two proteins that may promote cell survival are telomerase reverse transcriptase (TERT), an antisenescence protein,^10^, ^22^ and myocardin (MYOCD), a promyogenic transcription factor with anti‐apoptotic and pro‐angiogenic activities.^11^ The forced co‐expression of TERT and MYOCD, known to potentiate anti‐apoptotic, pro‐survival and pro‐angiogenic activities in AT‐MSCs,^23^, ^24^, ^25^ may improve CSC survival, by favouring their paracrine activities. The specific effects generated by the secretome from wild‐type AT‐MSC or AT‐MSC overexpressing TERT / MYOCD on the cardiac niche and their CSCs are an area so far unexplored of interesting investigation. Accordingly, here we investigated the potential of CSps and CSp‐derived CSCs to increase their resistance to simulated I/R (SI/R) and their commitment towards a cardiac or vascular lineage after preconditioning with AT‐MSC secretome overexpressing TERT and MYOCD, in the form of either conditioned media (CM) or extracellular vesicle (EV)‐enriched fraction.

## 2 METHODS

### 2.1 Animal care

All procedures were approved by the local Institutional Ethics Committee for Animal Research (Protocol number 11/2012/CEISA/COM). All studies conformed to the Guidelines from Directive 2010/63 EU of the European Parliament on the protection of animals used for scientific purpose of the United States National Institute of Health guidelines. A more detailed explanation of the methods reported below is provided in the Appendix S1.

### 2.2 Isolation and culture of adipose tissue‐derived mesenchymal stromal cells

Twelve‐month‐old male C57BL/6 mice (Charles River Laboratories) were anesthetized by inhalation of 2%‐5% isoflurane in oxygen and killed. Adipose tissue‐derived mesenchymal stromal cells (AT‐MSCs) were then isolated from the peri‐epididymal visceral adipose tissue by using a modified version of a previously described protocol, as described in the Appendix S1^19^ The vascular stromal fraction was plated, and AT‐MSCs selected based on their adherence to plastic. Before transduction, AT‐MSCs were cultured and subsequently characterized at both passages ≤3 and >3 to assess their expression of markers of MSCs, progenitor endothelial cells, pericytes, and smooth muscle cells.^24^

### 2.3 cDNA cloning and expression vector constructs of telomerase and myocardin

Full‐length cDNAs for human telomerase (TERT, 3.6 kb, Genebank accession number NM_198253.2) and human myocardin (MYOCD) isoform 1 (3.1 kb, Genebank accession number NM_153604.1) were amplified via polymerase chain reaction, subcloned and cloned into the pLenti‐TOPO cloning vector (Invitrogen), as previously described.^24^ A more detailed explanation of the methods reported below is provided in the Appendix S1.

### 2.4 Isolation of cardiospheres and cardiosphere‐derived cardiac stromal cells

One‐week‐old neonatal mice, 6‐week‐old adult mice and 1‐year‐old C57BL/6 mice were anesthetized by inhalation of 2‐5% isoflurane in oxygen and killed. Cardiac stromal cells (CSCs) were isolated from hearts trough the cardiosphere (CSp) isolation technique.^26^ A more detailed explanation of the methods reported below is provided in the Appendix S1.

### 2.5 Western analyses

Total protein extracts of CSCs and AT‐MSCs were isolated in ice‐cold radioimmunoprecipitation buffer (Sigma‐Aldrich). A more detailed explanation of the methods is provided in the Appendix S1.

### 2.6 Functional assessment by intracellular Ca^2+^ measurements of cardiospheres treated with adipose tissue‐mesenchymal stromal cell conditioned media

Intracellular Ca^2+^ fluxes were measured on AT‐MSCs [wild‐type or mock‐transduced AT‐MSCs or transduced with TERT and MYOCD (T/M AT‐MSCs)] and CSps, according to the protocol detailed in the Appendix S1. Before the analyses of intracellular Ca^2+^ fluxes, movies of cultured CSps (Appendix video) were recorded using a Nikon‐4500 digital camera connected to a Leica inverted microscope.

### 2.7 Isolation, characterization and fluorescent labelling of extracellular vesicles from adipose tissue‐mesenchymal stromal cell culture supernatants

Mock‐transduced AT‐MSCs or T/M AT‐MSCs used to produce an extracellular vesicle (EV)‐enriched fraction were grown at a concentration of 4 × 10^7^ in 175 cm^2^ flasks. Prior to isolation, cells were washed three times with phosphate buffered saline, and EV production could take place for 72 hours in serum‐free Dulbecco's modified Eagle medium (DMEM) to avoid contamination of EVs already present in foetal bovine serum. Cell viability under serum‐free conditions was found to be >90%‐95%. The conditioned medium containing EV was transferred to 50‐mL centrifuge tubes (Thermo Fisher Scientific) and centrifuged at 2500 × *g* at 4°C for 5 minutes to remove cells and cellular debris. Isolation of the EV‐enriched fraction by ultrafiltration and fluorescent labelling of EVs was performed as described in the Appendix S1. EV‐enriched fraction quantitation and size distribution analyses were performed by nanoparticle tracking analysis (Figure S1). EV‐enriched fractions were further characterized by the lipid‐to‐protein ratio (Figure S1) and immunoblotting for the expression of CD63 and ALG‐2‐interacting protein X (ALIX).^23^

### 2.8 Analysis of adipose tissue‐mesenchymal stromal cell‐induced cardiac stromal cell cytoprotection in simulated ischaemia/reperfusion

The cytoprotective effect of AT‐MSCs on CSCs was tested in CSCs subjected to the previously described SI/R experiments, with cycles of hypoxia‐reoxygenation (Figure S2).^27^ CSCs were first preconditioned by culturing them for 72 hours in CM or EV‐enriched fraction at 3 different concentrations (0.1, 1.0 and 10 mg/mL) and harvested from 72 hours cultures of mock‐transduced AT‐MSCs or T/M AT‐MSCs. Afterwards, preconditioned CSCs were subjected to SI/R or normoxic conditions for 2.5 hours at 37°C, and cell viability was measured by the Trypan blue assay. In normoxic conditions, the culture medium was replaced with a normoxic solution (in mM: NaCl 125, KCl 5.4, NaH_2_PO_4_ 1.2, MgCl_2_ 0.5, HEPES 20, glucose 15, taurine 5, CaCl_2_ 1, creatine 2.5, BSA 0.1%, pH 7.4, 310 mOsm/L) and cells were incubated in a normoxic incubator at 37°C for 2.5 hours. To simulate ischaemic conditions, cells were incubated in a hypoxic solution (in mM: NaCl 119, KCl 5.4, MgSO_4_ 1.3, NaH_2_PO_4_ 1.2, HEPES 5, MgCl_2_ 0.5, CaCl_2_ 0.9, Na‐lactate 20, BSA 0.1%, 310 mOsm/L, pH = 6.4) and exposed to a constant flow of a mixture of 95% N_2_ and 5% CO_2_ for 2.5 hours at 37°C. Either normoxic or SI treatments were followed by 2.5 hours of treatment with differentiating medium and in a 37°C incubator with 95% air and 5% CO_2_ prior to harvesting. A more detailed explanation of the methods is provided in the Appendix S1.

### 2.9 Statistical analysis

Data are expressed as mean ± standard deviation (SD). Two‐group comparisons were performed by using the Student *t* test for unpaired values. Multiple‐group comparisons were performed by using analysis of variance and the Gabriel or Tukey Honestly Significant Difference (HSD) post hoc tests to determine statistical significance within and between groups. *P* values <.05 were considered statistically significant.

## 3 RESULTS

### 3.1 Adipose tissue‐mesenchymal stromal cells overexpressing TERT and MYOCD and their secretome improve spontaneous intracellular calcium changes of aged cardiospheres in vitro

**FIGURE 1:**
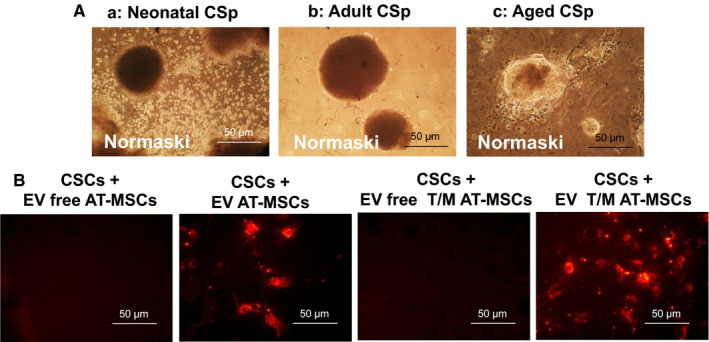
A, Representative images of cardiospheres (CSps), observed in Nomarski interference contrast, isolated from hearts of neonatal (a), 6‐month‐old (b) and 1‐year‐old (c) C57BL/6 mice. B, EV‐enriched fraction taken up by cardiosphere‐derived stromal cells (CSCs). PKH26‐stained EV‐enriched fraction (red) harvested from conditioned media of mock‐transduced adipose tissue‐derived mesenchymal stromal cells (AT‐MSCs) or AT‐MSCs overexpressing TERT and MYOCD (T/M‐MSCs) are taken up by CSCs within 72 h of treatment

**FIGURE 2:**
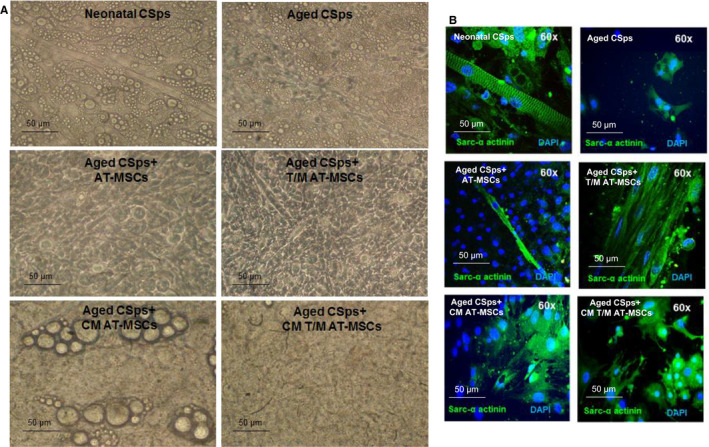
In vitro effects of AT‐MSCs overexpressing TERT and MYOCD and their secretome on sarcomeric α‐actinin expression in CSps from aged (1‐year‐old) C57BL/6 mice. A, Representative images of CSps co‐cultured with AT‐MSCs acquired in Nomarski interference contrast. B, Representative confocal microscopy images of sarcomeric α‐actinin expression (green) in the cardiospheres (CSps), according to treatment groups. DAPI (blue) was used for nuclear counterstaining. Images were taken in the peripheral area of CSps

Interactions between AT‐MSCs and CSps were initially analysed in an in vitro co‐culture system. Young CSps obtained and cultured from neonatal, adult (6‐month‐old) and aged (1‐year‐old) C57BL/6 mice grew well and colonized in regular media and morphologically showed a growth pattern of typical stromal cells (Figure 1A). However, aged cells grew slower and formed smaller sizes of colonies. Fluorescence analysis of EV showed stronger signals from the PKH26 red fluorescence intracellular probe in EV‐expressing T/M AT‐MSCs co‐cultured with CSps (Figure 1B). This observation suggests an active secretome activity in the CSps/AT‐MSCs co‐culture, particularly in case of T/M co‐expression. Indeed, further features of young and old CSps co‐cultured with AT‐MSCs and T/M AT‐MSCs were revealed by bright‐field images, in terms of morphological alterations of cell differentiation (Figure 2A). Compared to cells from neonatal tissue, aged CSps appeared to have a low capacity to form elongated fibrocells with the appearance of cardiomyocytes. Immunofluorescent staining revealed that neonatal CSps expressed sarcomeric α‐actinin and had the sarcomeric striations typical of mature cardiomyocytes, whereas aged CSps were found to express sarcomeric α‐actinin without sarcomeric striations (Figure 2B). When aged CSps were co‐cultured with AT‐MSCs overexpressing TERT and MYOCD (T/M AT‐MSCs) or cultured in CM from T/M AT‐MSCs, they did not form elongated fibrocells (Figure 2A). Despite expressing sarcomeric α‐actinin, the protein did not show the typical sarcomeric striation pattern (Figure 2B).

**FIGURE 3:**
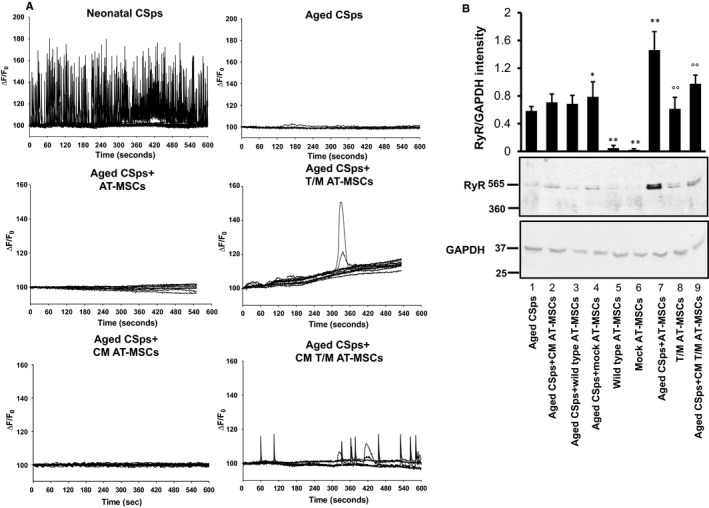
In vitro effects of AT‐MSCs overexpressing TERT and MYOCD and their secretome on intracellular Ca^2+^ spikes and ryanodine receptor levels in CSps from aged (1‐year‐old) C57BL/6 mice. A, Representative intracellular Ca^2+^ spikes in neonatal CSps, aged CSps, according to treatment groups. B, Representative Western blot showing the levels of ryanodine receptor (RyR) in aged CSps according to treatment groups. GAPDH levels were assessed as a loading control. Results presented as mean ± standard deviation, n = 3 independent experiments. *P < .05 aged CSps+mock AT‐MSCs vs aged CSps; **P < .01 wild‐type AT‐MSCs vs aged CSps; °°*P* < .01 T/M AT‐MSCs vs wild‐type AT‐MSCs or mock‐transduced AT‐MSCs; °°*P* < .01 aged CSps+CM T/M AT‐MSC vs aged CSps. Legend: CM, conditioned media; AT‐MSCs, mock‐transduced adipose tissue‐derived mesenchymal stromal cells; AT‐MSCs overexpressing TERT and MYOCD (T/M AT‐MSCs); GAPDH, glyceraldehyde 3‐phosphate dehydrogenase

Temporal analyses of spontaneous intracellular Ca^2+^ changes showed intense activity in neonatal CSps, whereas this activity was almost absent in aged CSps (Figure 3A). Aged CSps showed rare spontaneous Ca^2+^ spikes when co‐cultured with T/M AT‐MSCs, but they were almost quiescent when co‐cultured with mock‐transduced AT‐MSCs. Likewise, aged CSps showed isolated spontaneous Ca^2+^ spikes when conditioned with CM from T/M AT‐MSCs, but they did not show any significant activity when conditioned with CM from mock‐transduced AT‐MSCs (Figure 3A).

Western analysis showed that ryanodine receptor expression levels were detectable in aged CSps cultured alone and significantly increased when CSps were co‐cultured with T/M AT‐MSCs (vs aged CSps *P* = .01, n = 3 independent experiments) or cultured with CM from the T/M AT‐MSCs (vs aged CSps *P* = .03, n = 3 independent experiments). The protein level in mock‐transduced AT‐MSCs was much lower than that in the T/M AT‐MSCs (*P* = .0001, n = 3 independent experiments) (Figure 3B). The receptor level in aged CSps did not change significantly when cells were co‐cultured with mock‐transduced AT‐MSCs or cultured with CM from mock‐transduced AT‐MSCs.

### 3.2 The secretome of adipose tissue‐mesenchymal stromal cells overexpressing telomerase and myocardin and its extracellular vesicle‐enriched fraction exert cytoprotective effect on the cardiac stromal cells in an in vitro model of simulated ischaemia/reperfusion

**FIGURE 4:**
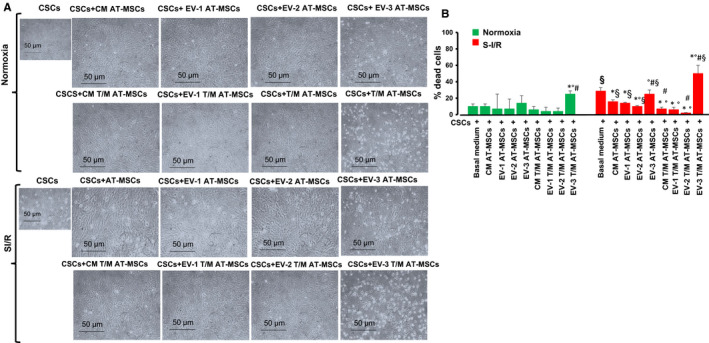
A, Representative microphotographs showing the effect of the secretome of AT‐MSC overexpressing TERT and MYOCD (T/M AT‐MSC) and its extracellular vesicle (EV)‐enriched fraction on viability of aged cardiosphere‐derived stromal cells (CSCs) from 1‐year‐old murine hearts in a simulated model of ischaemia/reperfusion (SI/R). The conditioned medium (CM) or EV‐enriched fraction were harvested from 72 h cultures of mock‐transduced AT‐MSCs or T/M AT‐MSCs. CM or EV‐enriched fraction were applied 72 h before simulated ischaemia/reperfusion (SI/R) or normoxia conditions. Round cells represent the proportion of cells detached from the surface of the plates in each culture conditions. B, Effect of treatments on cardiosphere‐derived stromal cells (CSCs) viability. Cell viability was measured with the Trypan blue assay. *P < .05 CM or EV‐treated vs basal medium CSCs; °*P* < .05 EV‐treated vs CM‐treated; ^#^ *P* < .05 CM or EV T/M AT‐MSCs‐treated vs CM or EV mock‐transduced AT‐MSCs; § < .05 vs normoxia

To assess how the secretome of T/M AT‐MSCs might affect CSCs during SI/R, we performed immunofluorescence, morphological and Western analyses. As shown above, we confirmed by immunofluorescence microscopy that AT‐MSC EVs are taken up by CSCs (Figure 1B). First‐passage CSCs from aged (1‐year‐old) C57BL/6 mice were preconditioned for 72 hours with one of the following treatments: (a) basal medium (control), (b) CM from mock‐transduced AT‐MSC; (c) CM from T/M AT‐MSCs; (d) the EV‐enriched fraction from mock‐transduced AT‐MSCs (tested at 0.1, 1.0 and 10 mg/mL); or (e) the EV‐enriched fraction from T/M AT‐MSCs (tested at 0.1, 1.0 and 10 mg/mL). CSCs were then exposed to normoxic or SI/R conditions (Figure 4). We found that CSCs cultured in the SI/R condition had significantly higher mortality than CSCs cultured in normoxic conditions (cell death: 28 ± 4 vs 10 ± 3%, *P* = .02, n = 3 independent experiments, 10 replicates each) (Figure 4A). However, when CSCs were pre‐treated with CM from mock‐transduced AT‐MSCs before being cultured in SI/R, cell mortality was decreased (% dead cells: 15 ± 2, *P* = .02). This decrease (cell salvage) was even more prominent when CSCs were pre‐treated with CM from the T/M AT‐MSCs (% dead cells: 7 ± 1, *P* = .01). Pre‐treatment with the EV‐enriched fraction from the mock‐transduced AT‐MSCs (% dead cells: 10 ± 1, *P* = .02) and T/M AT‐MSCs (% dead cells: 2 ± 1, *P* = .01) had varying effects, being concentration‐dependent. Only the low and intermediate concentrations of the EV‐enriched fraction (0.1 and 1 mg/mL) had a significant cytoprotective effect on CSCs, with the maximum effect seen with the 1 mg/mL EV‐enriched fraction. In contrast, the highest concentration of the EV‐enriched fraction from T/M AT‐MSCS increased cell death, suggesting a cytotoxic effect of the EV‐enriched fraction at 10 mg/mL.

**FIGURE 5:**
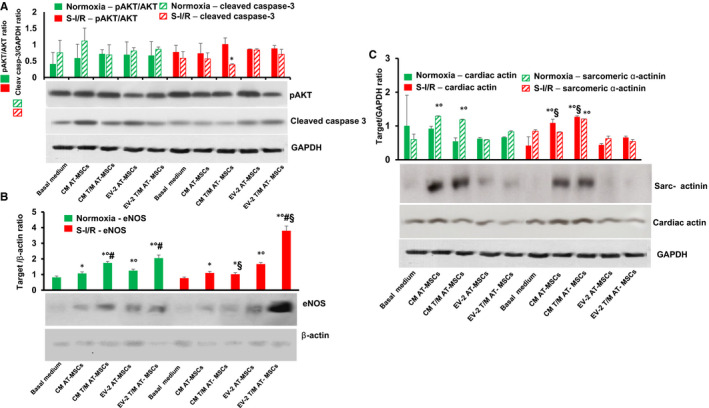
Effects of the secretome of AT‐MSC overexpressing TERT and MYOCD and their EV‐enriched fraction on aged CDCs in a simulated model of ischaemia/reperfusion. A‐C, Effect of treatments on the expression of cell survival (phosphorylated AKT) normalized for AKT and apoptosis (cleaved caspase‐3) markers A, endothelial marker (endothelial nitric oxide synthase, eNOS) B, and cardiomyocyte markers (cardiac actin and sarcomeric α‐actinin) C, in CSCs. Graphs are based on densitometric analysis of protein bands in Western blots. GAPDH levels were assessed as loading controls for cleaved caspase‐3, cardiac actin and sarcomeric α‐actinin, whereas beta‐actin levels were assessed as loading control for eNOS. Results presented are mean ± standard deviation, n = 3 independent experiments. A‐C:* *P* < .05 CM‐ or EV‐treated vs basal medium; °*P* <.05 EV‐treated vs CM‐treated; ^#^ *P* < .05 CM or EV T/M AT‐MSCs‐treated vs CM or EV mock‐transduced AT‐MSCs; ^§^ *P* <.05 SI/R vs normoxia. Legend: EV −1, extracellular vesicle‐enriched fraction 0.1 mg/mL; EV‐2, extracellular vesicle‐enriched fraction 1 mg/mL; EV‐3, extracellular vesicle‐enriched fraction 10 mg/mL

The findings reported above were corroborated by CSC molecular characterization. After CSCs were pre‐treated for 72 hours and then cultured in normoxic or SI/R conditions (as above), the total protein content of CSCs was collected and snap‐frozen for immunoblotting analyses. In cells exposed to SI/R, the level of pAKT (a pro‐survival marker) was consistent among all groups after normalization for constitutive AKT (*P* = .2, n = 3 independent experiments), whereas the level of cleaved caspase‐3 (an apoptosis marker) was significantly lower in CSCs treated with CM from T/M AT‐MSCs than in those cultured in control medium (*P* = .03, n = 3 independent experiments) (Figure 5A). In both the normoxic and the SI/R, pre‐treatment with CM or the 1 mg/mL EV increased eNOS expression (an endothelial cell marker) in CSCs (vs CSCs alone, *P* = .02, n = 3 independent experiments). In normoxic conditions, eNOS expression was higher in CSCs pre‐treated with CM from T/M AT‐MSCs compared with CM from mock AT‐MSCs, as well as higher in CSCs pre‐treated with EV from T/M AT‐MSCs or mock AT‐MSCs compared with CM from T/M AT‐MSCs or mock AT‐MSCs (*P* = .02, n = 3 independent experiments) (Figure 5B). The EV‐enriched fraction from the T/M AT‐MSCs induced even higher eNOS expression, particularly under SI/R conditions (*P* = .001, n = 3 independent experiments), indicating a possible synergistic effect between hypoxia and pro‐angiogenic paracrine factors contained in the EV‐enriched fraction of the AT‐MSCs.

An assessment of myocyte markers, such as cardiac actin and sarcomeric α‐actinin, showed a different pattern. The CM from mock‐transduced AT‐MSCs and T/M AT‐MSCs, but not the EV‐enriched fraction from these cells, increased the expression of cardiac actin and sarcomeric α‐actinin (*P* = .01, n = 3 independent experiments); there was no statistical difference between the effects of mock‐transduced AT‐MSCs and T/M AT‐MSCs (Figure 5C). These results indicate that the promyogenic factors of the secretome resided primarily in the EV‐free fraction.

## 4 DISCUSSION

We show here that overexpression of TERT and MYOCD increases survival of CSCs undergoing SI/R when preconditioned with either the culture media or the EV‐enriched fraction of culture media from AT‐MSCs. Moreover, T/M AT‐MSCs and their secretome improved spontaneous intracellular calcium changes and ryanodine receptor expression in aged CSps. This is the first demonstration of the cytoprotective effect of T/M AT‐MSCs on CSCs after SI/R. We here report that CSCs preconditioning with culture medium or its EV‐enriched fraction derived from T/M AT‐MSCs determines a better commitment of CSCs to a cardiovascular lineage. Preconditioning through the exposure to culture medium or the EV derived from AT‐MSCs here appears as ‘priming’ CSCs improving their survival and prompting their differentiation within the hostile microenvironment occurring during SI/R.^28^

Several preconditioning strategies before cell injection—pharmacological or genetic—have been previously described, increasing survival of injected cells into the hostile ischaemic tissue.^28^ Here, we tested the effects of jointly overexpressing antisenescence and anti‐apoptotic genes such as TERT and MYOCD, on survival and cardiovascular commitment of CSCs. MYOCD is a muscle transcription cofactor with anti‐apoptotic and vascular development activities.^29^ TERT plays a fundamental role in telomere length maintenance, cell survival, antisenescence and proliferation.^10^ The delivering TERT and MYOCD genes into MSCs from aged mice restores the function of these cells in vitro by increasing cell survival, proliferation, myogenic differentiation and pro‐angiogenic abilities.^22^, ^24^, ^30^ Quantitative proteomic approach based on 2DE and MALDI‐TOF/TOF mass spectrometry, show that overexpression of TERT and MYOCD can modulate the secretome composition of aged murine AT‐MSCs and improve their angiogenic function, suggesting that aged AT‐MSCs transduced with TERT and MYOCD become ‘rejuvenated’ in their paracrine activity.^23^

AT‐MSCs produce and release significant amounts of a host of humoral factors, such as growth factors and cytokines.^23^ In particular, T/M AT‐MSCs are capable of releasing large amounts of vascular endothelial growth factor (VEGF) and tissue inhibitors of metalloproteinases (TIMPs)^23^ in vitro, which mediate their pro‐angiogenic properties and cardiovascular cell commitment. In the present study, the EV‐enriched fraction from the T/M AT‐MSCs induced even higher eNOS expression in CSCs, and particularly in SI/R conditions, indicating a possible synergistic effect between hypoxia and pro‐angiogenic paracrine factors contained in the EV‐enriched fraction of AT‐MSCs.

EV has recently proposed as alternative therapeutic tool to cell transplantation in treatment of cardiovascular disease.^31^ Compared to cell therapy, EV is effective after systemic delivery.^32^ However, the optimization of protocols and cocktails to induce putative ‘cardiovascular’ gene expression programmes in vitro is the main challenges of this new therapy. There is considerable controversy over the type of reagents used in these protocols, ranging from cytokines, growth factors, however, showing conflicting and marginal results.^3^ In this regard, our report suggests a new strategy to increase the resistance of CSCs to SI/R and commitment to a cardiovascular lineage through cell preconditioning with CM or the EV‐enriched fraction from T/M AT‐MSCs.

Spontaneous cytosolic calcium fluctuations and oscillations have been reported in various tissues. Using confocal microfluorimetry, we showed spontaneous calcium transients in aged CSps exposed to T/M AT‐MSCs and their secretome, possibly due to the presence of intracellular calcium deposits that respond to muscarinic activation of cells reflecting expression of the ryanodine receptor. Calcium signalling plays essential roles in the development of the cardiovascular system. The effect of intracellular calcium transients on anti‐apoptotic gene expression has previously been demonstrated,^33^ which in immature cells might be a prerequisite for cell proliferation and differentiation. Dolmetsch et al^34^ reported that intracellular calcium oscillations in T lymphocytes increase both the efficacy and the information content of calcium signals that lead to gene expression and cell differentiation. We suggest that the spontaneous calcium transients in combination with the ryanodine receptor expression that we observed in our CSps exposed to T/M AT‐MSCs and their secretome may contribute to mechanisms involved in their differentiation.

We acknowledge that the present study has limitations. In vitro data obtained with the combination of CSCs and AT‐MSCs were not yet replicated in vivo with direct injection of the CM or EV‐enriched fraction from T/M AT‐MSCs. We have recently shown that transplantation of AT‐MSCs overexpressing MYOCD and TERT induced an increase in the pool of cardiac resident CSCs in the infarcted cardiac tissue in a murine model of acute myocardial infarction.^25^ Confirmation of these data also in a real I/R model will require further investigation. One additional limitation of this study is that it does not provide a complete characterization of the cardiovascular phenotype of CSCs after T/M AT‐MSC‐mediated preconditioning, nor it unravels signalling pathways involved in cardiovascular CSC commitment and resistance to hypoxia. Further studies will therefore add valuable insights into understanding how the T/M AT‐MSCs secretome influences CSCs in hypoxic conditions.

In conclusion, T/M AT‐MSCs, through a paracrine mechanism mediated by CM and EV, increase survival and promote a cardiovascular phenotype of CSCs undergoing SI/R. Thus, T/M AT‐MSCs appear to create a pro‐survival niche‐like microenvironment for CSCs undergoing SI/R. Further in vitro and in vivo studies are needed to clarify the specific mechanisms here involved.

## CONFLICT OF INTEREST

PF is founder and CEO of Pharmahungary Group, a group of R&D companies.

## AUTHOR CONTRIBUTIONS

**Rosalinda Madonna:** Conceptualization (lead); Data curation (equal); Formal analysis (equal); Funding acquisition (equal); Methodology (equal); Writing–original draft (equal); Writing–review and editing (equal). **Simone** **Guarnieri:** Data curation (equal); Formal analysis (equal); Methodology (equal); Writing–original draft (equal). **Csenger Kovácsházi:** Data curation (equal); Methodology (equal). **Aniko Gorbe:** Methodology (equal). **Zoltan** **Giricz:** Data curation (equal); Formal analysis (equal); Methodology (equal). **Yong Jian** **Geng:** Conceptualization (equal); Supervision (equal); Writing–original draft (equal). **Maria** **Addolorata Mariggio:** Data curation (equal); Methodology (equal); Validation (equal); Writing–original draft (equal). **Peter** **Ferdinandy:** Data curation (equal); Formal analysis (equal); Funding acquisition (equal); Methodology (equal); Supervision (equal); Validation (equal); Writing–original draft (equal). **Raffaele De Caterina:** Conceptualization (equal); Formal analysis (equal); Funding acquisition (equal); Supervision (equal); Validation (equal); Writing–original draft (equal).

## Supporting information

Fig S1

Fig S2

Appendix S1

## Data Availability

Data available on request from the authors
