# Supplementary figures and images for: Telomerase/myocardin expressing mesenchymal cells induce survival and cardiovascular markers in cardiac stromal cells undergoing ischaemia/reperfusion
Source: J Cell Mol Med. 2021 May 5;25(12):5381–90. doi: 10.1111/jcmm.16549 (PMC8184669; doi:10.1111/jcmm.16549)

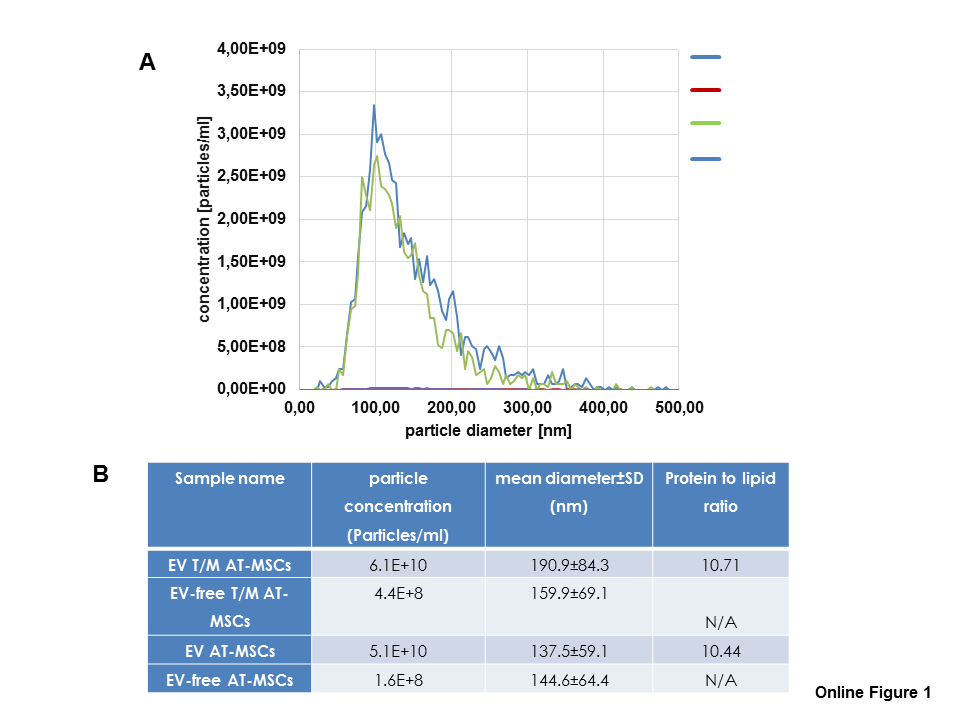

Supplement: Supplementary file 1 — Fig S1 [file JCMM-25-5381-s001.TIF]

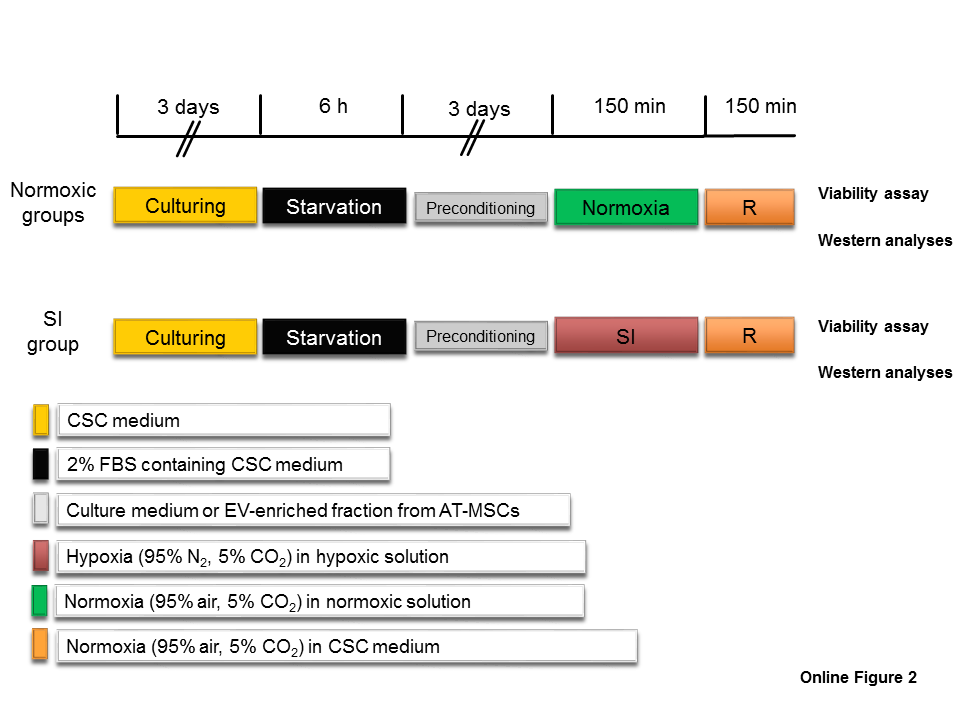

Supplement: Supplementary file 2 — Fig S2 [file JCMM-25-5381-s003.TIF]
