# Supplementary material for: Telomerase/myocardin expressing mesenchymal cells induce survival and cardiovascular markers in cardiac stromal cells undergoing ischaemia/reperfusion
Source: J Cell Mol Med. 2021 May 5;25(12):5381–90. doi: 10.1111/jcmm.16549 (PMC8184669; doi:10.1111/jcmm.16549)
Supplement: Supplementary file 3 — Appendix S1 [file JCMM-25-5381-s002.docx]

**Telomerase/Myocardin expressing mesenchymal cells induce survival and cardiovascular markers in cardiac stromal cells undergoing ischemia/reperfusion**

Rosalinda Madonna, MD, PhD, Simone Guarnieri, PhD, Csenger Kovácsházi, MSc, A. Görbe, MD, Zoltán Giricz, PharmD, PhD, Yong-Jian Geng, MD, PhD, Maria Addolorata Mariggiò, PhD, Peter Ferdinandy, MD, PhD, MBA,

and Raffaele De Caterina, MD, PhD

Center for Advanced Studies and Technology –CAST and Institute of Cardiology, "G. D'Annunzio" University, Chieti-Pescara, Chieti, Italy (S.G., M.A.M.); Department of Internal Medicine, McGovern School of Medicine, The University of Texas Health Science Center at Houston, Houston, Texas (R.M., Y.J.G.); Department of Neuroscience, Imaging and Clinical Sciences, “G. d’Annunzio” University, Chieti-Pescara and StemTeCh Group, Chieti, Italy (M.A.M., S.G.); Department of Pharmacology and Pharmacotherapy, Semmelweis University, Budapest, Hungary (Z.G., C.K, A.G., P.F.); and Pharmahungary Group, Szeged, Hungary (P.F. Z.G. A.G.); Department of Pathology, Cardiology Division, University of Pisa, Pisa, Italy (R.M., R.D.C.)

**SUPPLEMENTAL MATERIAL**

Correspondence to:

Raffaele De Caterina, MD PhD

Institute of Cardiology, University of Pisa

C/o Ospedale di Cisanello - Via Paradisa, 2, 56124 Pisa, Italy,

Tel: +39-050-315-2683

FAX: +39-050-315-2684

E-mail: [raffaele.decaterina@unipi.it](mailto:raffaele.decaterina@unipi.it)

Co-Corresponding author:

Rosalinda Madonna, MD PhD

Institute of Cardiology, University of Pisa

C/o Ospedale di Cisanello - Via Paradisa, 2, 56124 Pisa, Italy,

E-mail: [rosalinda.madonna@unipi.it](mailto:rosalinda.madonna@unipi.it)

###### DETAILED METHODS

**Isolation of adipose tissue-derived mesenchymal stromal cells and cell cultures**

To obtain the adipose tissue-derived mesenchymal stromal cells **(**AT-MSCs), the mice were anesthetized by isoflurane inhalation (2-5% isoflurane in oxygen) and then euthanized. The AT-MSCs were isolated from the peri-epididymal visceral adipose tissue of each mouse according to a modified version of a protocol originally described by Zuk and colleagues ^1^. Briefly, adipose tissue was minced mechanically and digested with collagenase. After adipocyte removal, the vascular stromal fraction was plated at a density of 1,000 cells/cm^2^ in Dulbecco’s Modified Eagle Medium (DMEM, GIBCO, Invitrogen, Carlsbad, CA) supplemented with L-glutamine (2 mmol/L), penicillin (100 U/mL), streptomycin sulfate (100 μg/mL), nonessential amino acids (0.1 mmol/L), 2‑mercaptoethanol (1 mmol/L), and 20% fetal bovine serum. After 24 hours, the AT-MSCs were selected based on their plastic adherence properties, and the nonadherent cells were removed. Before transduction, AT-MSCs were cultured in 100-mm dishes at high density and subsequently characterized at passages ≤ 3 and at passages > 3 to assess their expression of markers for mesenchymal stem cells, endothelial progenitor cells, pericytes, and smooth muscle cells, as previously described by flow cytometry ^2^.

**Cloning of telomerase reverse transcriptase and myocardin lentiviral expression plasmids**

Total RNA was isolated by using a Qiagen RNA isolation kit and was reverse transcribed directly into single-stranded cDNA by using the SuperScript™ III Platinum® One-step RT-PCR System (Invitrogen) according to manufacturer’s protocol. Full-length cDNAs for human telomerase (TERT, 3.6 kb) and human myocardin (MYOCD) isoform 1 (3.1 kb) were amplified by means of PCR and subcloned into the pLenti-TOPO cloning vector (Invitrogen), as detailed previously ^2^. Briefly, the primers used for PCR amplification of murine TERT were 5’-CACCATGACCCGCGCTCCT-3’ (forward) and 5’-CGCCCAGTCCAAAATGGTCTG-3’ (reverse); the primers used for the PCR amplification of murine MYOCD were 5’-CACCATGACACTCCTGGGGTCTGAAC- 3’ (forward) and 5’GTCCCACTGCTGTAAGTGGAGATCCAT-3’ (reverse). The full-length TERT and MYOCD cDNA sequences, as well as the constructs, were confirmed by using restriction enzyme digestion analysis and DNA sequencing.

For lentiviral production, all cell culture procedures were performed under biosafety level 2 conditions and following previously described procedures ^2^. The genome of the HIV-1-based lentivirus consisted of the plasmid of interest and 3 packaging plasmids (pLP1, pLP2, and pLP/VSVG), which supply the helper functions and the structural and replication proteins required to produce the lentivirus. The plasmid of interest encoded the yellow fluorescent protein (YFP)-TERT or V5-MYOCD fusion proteins under the control of a cytomegalovirus promoter (CMV). To enhance the level of gene expression, we used a lentiviral vector that contained a spleen-focus forming virus (SFFV)-LTR promoter and a woodchuck hepatitis post-transcriptional regulatory element ^2^. To obtain lentiviral particles, 293FT cells were plated 24 hours before transfection in 20-mm dishes in growth medium (10% FCS, high-glucose DMEM) that did not contain antibiotics; the cells were sub-confluent. Forty micrograms of plasmid DNA was used for transfection. To achieve precipitation, plasmids were diluted with 100 μL of Opti-MEM and 120 μL of Lipofectamine 2000 to a final volume of 220 μL and incubated at room temperature for 20 minutes. The DNA/Lipofectamine 2000 complex was added dropwise to culture dishes and then incubated at 37° C in a 5% CO_2_ incubator overnight. After 24 hours, the medium was replaced with 20 mL of 10% high-glucose DMEM; this conditioned medium was collected after an additional 48 hours of incubation and filtered through a 0.45 μm cellulose acetate filter. To concentrate the pLenti-CMV-YFP/TERT and pLenti-CMV-V5/MYOCD vectors, 1 mL of medium containing the lentiviral vector was transferred to a 1.7-mL microcentrifuge tube and spun in a bench microcentrifuge at 18,000 g for 1 hour at 4°C. The supernatant was then carefully removed from the tube. The pellet was gently resuspended into 100 μL of DMEM. The multiplicity of infection (MOI) was determined in 293T cells and AT-MSCs by using serial dilutions of the vector stocks. AT-MSCs (2x10^5^ cells/well) were exposed to serial dilutions of the following vectors for 5 days: pLenti-CMV-V5 (mock), pLenti-CMV-MYOCD-V5, pLenti-CMV-YFP (mock), or pLenti-CMV-TERT-YFP; the serial dilutions corresponded with MOIs ranging from 1 to 40. Cells were infected with 50 μL (MOI=1), 100 μL (MOI=10), 200 μL (MOI=20), or 400 μL (MOI=40) of the concentrated vector. All of the in vivo and in vitro assays were performed at the infection level capable of producing the maximum efficiency of transduction while producing no cytotoxic effects.

**Fluorescence-activated cell sorting and western analysis of transduced cells**

Murine AT-MSCs (1x10^6^ cells) were plated in 60-mm culture dishes. Cells were incubated with serial dilutions of concentrated lentivirus stock for 16 h in a volume of 10 mL in the presence of polybrene (16 μg/mL). Cells were cultured for 5 days, trypsinized, and analyzed for YFP-TERT expression with a BD FACS Canto II (BD Biosciences, San Jose, CA) equipped with a 488-nm laser (for excitation of YFP, 517 nm). Data were analyzed by using Fluorescence-activated cell sorting (FACS) Diva software (BD Biosciences). The percentage of YFP^+^ cells indicated the percentage of transduced cells. MYOCD-V5 expression was analyzed by Western blot.

**Isolation of cardiospheres and cardiosphere-derived stromal cells**

Murine tissue was derived from hearts (1 week-old neonatal mice, 6 weeks-old adult mice and 1 year-old aged mice) of male C57BL/6 mice (Charles River Italia, Lecco, Italy). Isolated myocardial tissue was cut into 1 mm pieces, washed with Ca-Mg-free phosphate-buffered solution (PBS) (Invitrogen), and digested for 5 minutes at 37°C with 0.2% trypsin (Invitrogen). The obtained cells were discarded, and the remaining tissue fragments were washed with complete explant medium (CEM) (Iscove’s Modified Dulbecco’s Medium [IMDM] supplemented with 10% fetal calf serum, 100 U/mL penicillin G, 100 μg/mL streptomycin, 2 mmol/L L-glutamine, and 0.1 mmol/L 2-mercaptoethanol) and cultured as explants in fibronectin-coated plates in CEM at 37°C and 5% CO2. After a period ranging from 1 (neonatal) to 3 (adult and aged) weeks, a layer of fibroblast-like cells as generated from adherent explants over which small, phase-bright cells migrated. These phase-bright cells were collected by pooling two washes with Ca-Mg-free PBS, one wash with 0.53 mmol/L EDTA (Versene, Invitrogen) (1 to 2 minutes), and one wash with 0.5 g/L trypsin and 0.53 mmol/L EDTA (Invitrogen) (2 to 3 minutes) at room temperature. 4×10^5^ cells were seeded in poly-D-lysine-coated 24 multi-well plates (BD Bioscences, Milan, Italy) in cardiosphere (CSp) growing medium (CGM) (35% complete IMDM/65% DMEM–Ham F-12 mix with antibiotics and L-Glu, as in CEM), containing cocktail of growth factors including 2% B27, 0.1 mmol/L 2 mercaptoethanol, 10 ng/mL epidermal growth factor [EGF], 20 ng/mL basic fibroblast growth factor [bFGF], 40 nmol/L cardiotrophin-1, 40 nmol/L thrombin. The first passage of cardiac stromal cells (CSCs) from neonatal and aged mice were used for the experiments as indicated below. For cryopreservation, we used CEM/DMEM–Ham F12 at 50:50, 5% B27, and 10% DMSO as the freezing medium.

**Isolation and fluorescent labeling of extracellular vescicle - enriched fraction** **from adipose tissue-derived mesenchymal stromal cell culture media**

Mock-transduced AT-MSCs or TERT/MYOCARDIN overexpressing AT-MSCs (T/M AT-MSCs) used for producing extracellular vescicle (EV) enriched fraction or conditioned medium (CM) were grown at a concentration of 4×10^7^ in 175 cm^2^ flasks. Prior to isolation, cells were washed 3 times with PBS and cultured for 72 h in serum-free DMEM medium for collecting EV-enriched fraction. Serum free media was used to avoid contamination of the preparations with EV-enriched fraction present in fetal bovine serum. Cell viability under serum-free conditions was found to be > 90-95%. The conditioned medium was transferred to 50-mL centrifuge tubes (Thermo Fisher Scientiﬁc, Rockford, IL) and centrifuged at 2500×g, at 4°C for 5 min to remove cells and cellular debris. Isolation of EV-enriched fraction was performed, as described below. The CM was carefully removed, subsequently transferred to new 50-mL centrifuge tubes and filtered by gravity through a 0.8 µm filter (Millipore, Billerica, MA, cat. Number SLAA033SS), then submitted to a centrifugation at 13,500×g, at 4°C for 40 min. The supernatant resulting from centrifugation was carefully removed, placed in Amicon Ultra-15 ultrafiltration units with Ultracel-100 membrane (Millipore, cat. Number UFC910024) and further centrifuged at 4000×g, at 4 °C for 15 min. Either the filtered (EV-free fraction) and retentate (EV-enriched fraction) from the ultrafiltration units were collected and used immediately or stored at -70 °C until use. Recovered protein contents from EV-free and EV-enriched fraction were determined using the Pierce BCA protein assay kit (Thermo Fisher Scientiﬁc) per manufacturer’s protocol. EV-enriched fraction were labeled with PKH26 (Sigma-Aldrich, St Louis,MO) according to manufacturer’s protocol. Briefly, 7.5 mg of EV enriched fraction were resuspended in 1 mL of diluent C (Sigma-Aldrich) and mixed with PKH26 diluted in Diluent C for a final concentration of 2x10^-6^ M PKH26. The EV- enriched fraction dye suspension was incubated for 5 min with regular mixing. Excess dye from the labeled EV enriched fraction were removed by washing in PBS for 3 times. A mixture without EV enriched fraction was used as a negative control.

**Characterization of extracellular vescicle - enriched fraction** **from adipose tissue-derived mesenchymal stromal cell culture media by Nanoparticle tracking analysis and protein to lipid ratio measurement**

Amount and size distribution of particles was measured by Nanoparticle tracking analysis (NTA) using ZetaView® PMX-120 (Particle Metrix, Inning am Ammersee, Germany). Samples were diluted 1000-fold with 0.1 µm-filtered PBS. All NTA measurements were performed with a gain of 33.6, a shutter value of 100 (corresponding to an exposure time of 10 ms) and a frame rate of 30 frames per second.

To enable protein and lipid measurements, samples were concentrated with ultracentrifugation for 2h at 100.000×g, then extracellular vescicle (EV)**-**enriched fraction pellets were resuspended in PBS. Protein concentration of EV**-**enriched fractions were measured by BCA kit (Thermo Fisher Scientific, Waltham, MA USA) according to the instructions of the manufacturer with BSA as a standard. Lipid content of EV enriched fractions was measured according to Visnovitz et al.^5^. Briefly, 200 µL of 96% sulphuric acid (Molar Chemicals) was added either to 40 µL of liposome standards or to 40 µL EV**-**enriched fractions suspended in PBS and incubated at 90°C for 20 min. Tubes were cooled and 120 µL of phospho-vanillin reagent was added to each tube. Next, 280 µL of sample was transferred to a 96 well plate (Thermo) and incubated for 1 h at 37°C. Absorbance at 540 nm was determined with a plate reader (Multiskan MS, Labsystems). As a standard an aqueous phase liposome of 1,2-Dioleoyl-sn-glycero-3-phosphocholine was used.

**Western analyses**

Total protein extracts of CSCs in each experimental group were isolated in ice-cold radioimmunoprecipitation buffer (Sigma Aldrich)***.*** The proteins were separated under reducing conditions and electroblotted onto a polyvinylidene fluoride membrane (Immobilon-P; Millipore, Bedford, MA). The membranes were blocked with 3% milk in TBS-Tween (50 mM tris, 150 mM NaCl, 0.05% Tween-20), and then incubated overnight at 4°C with primary antibodies against one of the following: (1) cardiac actin (Sigma Aldrich; 1:5000 dilution); (2) endothelial nitric oxide synthase (eNOS, BD Transduction Laboratories, Franklin Lakes, NJ, 1:2500 dilution); (3) pAKT and AKT (Cell Signaling, 1:1000 dilution); (4) cleaved caspase-3 (Cell Signaling, Danver, MA, 1:1000); (5) alpha sarcomeric actinin (Sigma, 1:5000). Equal loading and protein transfer were verified by stripping and reprobing each blot with antibodies against GAPDH or β-actin (Sigma).

**Extracellular vesicle-enriched fraction** **uptake by cardiac stromal cells and analysis of adipose tissue-derived mesenchymal stroma cell-induced cardiac stromal cell cytoprotection and differentiation in simulated ischemia/reperfusion**

The cytoprotective effect of AT-MSCs on CSCs was tested in the model of simulated ischemia/reperfusion (SI/R) ^6^. CSCs were first preconditioned by culturing them for 72 h in CM or EV-enriched fraction at three different concentrations (0.1, 1.0, and 10 mg/mL) harvested from 72 h cultures of mock-transduced AT-MSCs or T/M AT-MSCs. As EV-enriched fraction were previously PKH26-labeled, their uptake by CSCs was verified by localization of red fluorescence in the CSCs under fluorescence microscope. Afterwards, preconditioned CSCs were subjected to SI/R or normoxic conditions for 2.5 h at 37^o^C; and cell viability was measured by trypan blue assay. In normoxic conditions, the culture medium was replaced with a normoxic solution (in mM: NaCl 125, KCl 5.4, NaH_2_PO_4_ 1.2, MgCl_2_ 0.5, HEPES 20, glucose 15, taurine 5, CaCl_2_ 1, creatine 2.5, BSA 0.1%, pH 7.4, 310 mOsm/l) and cells were incubated in a normoxic incubator at 37°C for 2.5 h. For ischemic conditions, cells were incubated in hypoxic solution (in mM: NaCl 119, KCl 5.4, MgSO_4_ 1.3, NaH_2_PO_4_ 1.2, HEPES 5, MgCl_2_ 0.5, CaCl_2_ 0.9, Na-lactate 20, BSA 0.1%, 310 mOsm/l, pH=6.4) and exposed to a constant flow of a mixture of 95% N_2_ and 5% CO_2_ for 2.5 hours at 37°C. The following groups were set: 1. CSCs cultured in the control media and then subjected to normoxic conditions; 2. CSCs+ cultured in the CM from mock-transduced AT-MSCs and then subjected to normoxic conditions; 3. CSCs+ cultured in the CM from T/M AT-MSCs and then subjected to normoxic conditions; 4. CSCs+ cultured in the EV-enriched fraction at concentration 1 (low concentration: 0.1 mg/mL) from mock-transduced AT-MSCs and then subjected to normoxic conditions; 5. CSCs+ cultured in the EV-enriched fraction at concentration 2 (intermediate concentration: 1.0 mg/mL) from mock-transduced AT-MSCs and then subjected to normoxic conditions; 6. CSCs+ cultured in the EV-enriched fraction at concentration 3 (high concentration: 10 mg/mL) from mock-transduced AT-MSCs and then subjected to normoxic conditions; 7. CSCs+ cultured in the EV-enriched fraction at concentration 1 (low concentration: 0.1 mg/mL) from T/M AT-MSCs and then subjected to normoxic conditions; 8. CSCs+ cultured in the EV-enriched fraction at concentration 2 (intermediate concentration: 1.0 mg/mL) from T/M AT-MSCs and then subjected to normoxic conditions; 9. CSCs+ cultured in the EV -fraction at concentration 3 (high concentration: 10 mg/mL) from T/M AT-MSCs and then subjected to normoxic conditions; 10. CSCs cultured in the control media and then subjected to hypoxic conditions; 11. CSCs+ cultured in the CM from mock-transduced AT-MSCs and then subjected to hypoxic conditions; 12. CSCs+ cultured in the CM from T/M AT-MSCs and then subjected to hypoxic conditions; 13. CSCs+ cultured in the EV-enriched fraction at concentration 1 (low concentration: 0,1 mg/mL) from mock-transduced AT-MSCs and then subjected to hypoxic conditions; 14. CSCs+ cultured in the EV-enriched fraction at concentration 2 (intermediate concentration: 1.0 mg/mL) from mock-transduced AT-MSCs and then subjected to hypoxic conditions; 15. CSCs+ cultured in the EV-enriched fraction at concentration 3 (high concentration: 10 mg/ml) from mock-transduced AT-MSCs and then subjected to hypoxic conditions; 16. CSCs+ cultured in the EV-enriched fraction at concentration 1 (low concentration: 0.1 mg/mL) from T/M AT-MSCs and then subjected to hypoxic conditions; 17. CSCs+ cultured in the EV-enriched fraction at concentration 2 (intermediate concentration: 1.0 mg/mL) from T/M AT-MSCs and then subjected to hypoxic conditions; 18. CSCs+ cultured in the EV-enriched fraction at concentration 3 (high concentration: 10 mg/mL) from T/M AT-MSCs and then subjected to hypoxic conditions. Either normoxic or SI/R treatments were followed by 2.5 h of treatment with CSC medium, and in a 37°C incubator with 95% air and 5% CO_2_ prior to harvest. After trypan blue analyses, total protein extracts from cells in each experimental group were isolated in ice-cold radioimmunoprecipitation buffer and immunoblotted for western analysis of the following markers: endothelial (endothelial nitric oxide synthase, eNOS), cell survival (pAKT and AKT) and apoptosis (cleaved caspase 3), and cardiomyocyte (cardiac actin and alpha sarcomeric actinin) markers. Beta-actin or GAPDH were used as internal control.

**Intracellular Ca^2+^ measurements**

Both non-excitable and excitable cells, like muscle cells, are characterized by the presence of spontaneous intracellular Ca^2+^ variations that coordinate and regulate many cellular activities ^3,4^. For in vitro experiments testing the effects of AT-MSCs (wild-type, mock transduced or overexpressing TERT and MYOCD) on contractile function of endogenous CSp, intracellular Ca^2+^ transients were analyzed and contractile activity was recorded (Appendix video).

Analyses were performed on neonatal CSp harvested from 1-week-old mouse hearts, and “aged” CSps harvested from 1-year-old mouse hearts. Neonatal and aged CSps were formed and maintained in CGM containing cardiomyogenic cocktail (as described in the cell culture section) at 37°C and 5% CO_2_, until the experiments. After 72 h and before the intracellular Ca^2+^ measurements, CGM was replaced with either non-conditioned control medium (CM: DMEM, supplemented with 2 mmol/L L-glutamine, 100 U/mL penicillin, 100 μg/mL streptomycin sulfate, 0.1 mmol/L non-essential amino acids, 1 mmol/L 2‑mercaptoethanol, in serum-free conditions) or CM harvested from 72 h cultures of wild-type AT-MSCs, mock-transduced AT-MSCs or T/M AT-MSCs. In parallel experiments, CSps from 1-year-old mouse hearts were directly co-cultured in control medium from three day -culture with wild-type AT-MSCs, mock-transduced AT-MSCs or T/M AT-MSCs. In additional control experiments, AT-MSCs (wild-type, mock-transduced or overexpressing TERT and MYOCD) were cultured under the same conditions without CSps. Before the analyses of intracellular Ca^2+^ transients, movies of cultured CSps (Appendix video) were recorded using a Nikon-4500 digital camera connected to a Leica inverted microscope. Intracellular Ca^2+^ fluxes were measured using the calcium sensitive dye Fluo4-acetoxymethyl ester (Fluo4/AM, Life Technologies, Monza, Italy) that is cleaved by intracellular esterases to give rise to a Ca^2+^ sensitive fluorescence probe, with a Zeiss Axio Examiner Upright Microscope (Carl Zeiss, Jena, Germany) equipped with a 20X 1.0 NA water immersion objective connected by an optical fiber to a 75W Xenon lamp and a monochromator (OptoScan, Cairn Instrument, UK, England) with sub-millisecond band-pass and wavelength controls and back-illuminated Electron Multiplying Charge Coupled Device (EMCCD) camera (Evolve 512, Photometrics, Tucson, USA). Cell samples were incubated with 5 µM Fluo-4/AM in normal external solution (NES, in mM: 140 NaCl, 2.8 KCl, 2 CaCl_2_, 2 MgCl_2_, 10 Glucose, 10 Hepes, pH 7.3) supplemented with 1% (w/v) bovine serum albumin, at 37 °C for 40 min. After washing, Fluo4-loaded cells were maintained in NES and excited at 488 nm, fluorescence images were acquired at 5 frames/s with an EMCCD camera and stored on an interfaced computer for off-line analysis. Temporal changes in intracellular Ca^2+^ were calculated as mean fluorescence intensity signal in a selected cell area as (f-f0/f0)x100, where f is the fluorescence emission of a single loaded cell acquired during a time lapse and f0 is the mean fluorescence intensity value of the same cell calculated from the first image. After analyses of intracellular Ca^2+^ levels, CSps were stained by direct immunofluorescence for the expression of sarcomeric α-actinin using FITC-conjugated mouse monoclonal anti-sarcomeric α-actinin antibody (Sigma, 1:500 dilution). Total protein extracts from CSps in each experimental group were isolated in ice-cold radioimmunoprecipitation buffer and immunoblotted for Western blot analysis of ryanodine receptor (RyR), which was detected by primary antibody against RyR-1 and RyR-2 isoforms (monoclonal mouse IgG1 clone, Invitrogen, 1:5000 dilution). Equal loading and protein transfer were verified by stripping and reprobing each blot with antibodies against GAPDH or beta-actin (Sigma).

**RESULTS**

**Quantitative and qualitative analysis of adipose tissue-derived mesenchymal stromal cell** **extracellular vesicles enriched fractions**

To investigate size distribution and qualitative properties of extracellular vesicles (EV)-enriched fraction isolated from adipose tissue-derived mesenchymal stromal cells (AT-MSCs) overexpressing telomerase (TERT) and myocardin (MYOCD) or wild type AT-MSCs, we performed nanoparticle tracking analysis. EV-enriched fractions isolated from T/M AT-MSCs contained a high number of particles, which was comparable to that of the EV-enriched fractions isolated from wild type AT-MSCs (6.1E+10/ml vs. 5.1E+10/ml). Size distribution of EV-enriched fraction from T/M AT-MSCs was closely correlating to that of EV-enriched fraction wild-type AT-MSCs indicating no major difference in EV composition. EV-depleted samples contained approximately 100-fold less particles, (4.4E+8/ml vs. 1.6E+8/ml) evidencing efficient EV removal from the samples (Appendix Figure 1, A).

To further characterize the purity of EV-enriched fractions, protein and lipid contents were measured. While EV-enriched fractions contained comparable amounts of lipids, lipid content of EV-depleted samples were below detection limit, which confirmed the NTA measurements, and indicated that the vast majority of EVs were removed from the Exo-free samples. The protein to lipid ratio of our EV samples (Appendix Figure 1, B) correlates well with data obtained from small EV samples isolated from cardiac cell lines, as published by Visnovitz et al. ^5^, which indicates that the purity of EV samples are comparable to that of the EV samples isolated from cell culture supernatants via widely accepted, ultracentrifugation-based methods. These data showed that EV-enriched fractions isolated from AT-MSCs overexpressing TERT and MYOCD and wld-type AT-MSCs contained comparably high concentration of particles and that EV-depleted samples contained an about 100-fold less particles as compared to the EV-enriched fractions. Protein to lipid ratio of particles were comparable with reference values of EVs isolated from cell cultures with the gold standard ultracentrifugation-based method suggesting adequate purity.

**REFERENCES**

1. Zuk PA, Zhu M, Mizuno H, et al. Multilineage cells from human adipose tissue: implications for cell-based therapies. Tissue Eng 2001;7:211-228.

2. Madonna R, Taylor DA, Geng YJ, et al. Transplantation of mesenchymal cells rejuvenated by the overexpression of telomerase and myocardin promotes revascularization and tissue repair in a murine model of hindlimb ischemia. Circ Res 2013;113:902-914.

3. Lee YS, Liu OZ, Sobie EA. Decoding myocardial Ca(2)(+) signals across multiple spatial scales: a role for sensitivity analysis. J Mol Cell Cardiol 2013;58:92-99.

4. Dolmetsch RE, Xu K, Lewis RS. Calcium oscillations increase the efficiency and specificity of gene expression. Nature 1998;392:933-936.

5. Visnovitz T, Osteikoetxea X, Sodar BW, et al. An improved 96 well plate format lipid quantification assay for standardisation of experiments with extracellular vesicles. J Extracell Vesicles 2019;8:1565263.

6. Chen T, Vunjak-Novakovic G. In vitro Models of Ischemia-Reperfusion Injury. Regen Eng Transl Med 2018;4:142-153

**LEGEND TO ONLINE FIGURES**

**Appendix Figure 1: Characterization of adipose tissue-derived mesenchymal stromal cell EV-enriched fractions. (A) Nanoparticle tracking analysis measurement of EV samples.** Nanoparticle tracking analysis (NTA) confirmed that samples contained EVs in with peak diameters of approximately 100 nm. Particle concentrations are comparable in adipose tissue-derived mesenchymal stromal cells overexpressing TERT and MYOCD (T/M AT-MSCs) and AT-MSC samples. Exo-free samples contained negligible amount of particles. **(B)** Tabular data of NTA and protein to lipid measurements.

**Appendix Figure 2: Experimental protocol of preconditioning and simulated ischemia/reperfusion on cardiac stromal cells.** After preconditioning and SI/R, cell’s viability was measured by trypan brue assay. Expression of vascular (eNOS) and cardiac (cardiac actin, sarcomeric α-actinin) markers, and marker of apoptosis (cleaved caspase-3) and survival (pAKT, AKT) was measured by western analyses.

**Appendix video**: Time lapse of spontaneous contractile activity in neonatal cardiospheres isolated from hearts C57BL/6 mice.
